# Exploring Physical and Chemical Factors Influencing the Properties of Recombinant Prion Protein and the Real-Time Quaking-Induced Conversion (RT-QuIC) Assay

**DOI:** 10.1371/journal.pone.0084812

**Published:** 2014-01-03

**Authors:** Keding Cheng, Angela Sloan, Kristen M. Avery, Michael Coulthart, Michael Carpenter, J. David Knox

**Affiliations:** 1 National Microbiology Laboratory, Public Health Agency of Canada, Winnipeg, Manitoba, Canada; 2 Department of Human Anatomy and Cell Sciences, Faculty of Medicine, University of Manitoba, Winnipeg, Manitoba, Canada; 3 Centre for Food-borne, Environmental and Zoonotic Infectious Diseases, Public Health Agency of Canada, Ottawa, Ontario, Canada; 4 Department of Medical Microbiology, Faculty of Medicine, University of Manitoba, Winnipeg, Manitoba, Canada; University of Melbourne, Australia

## Abstract

Real-time quaking-induced conversion (RT-QuIC), a highly specific and sensitive assay able to detect low levels of the disease-inducing isoform of the prion protein (PrP^d^) in brain tissue biopsies and cerebral spinal fluid, has great potential to become a method for diagnosing prion disease *ante mortem*. In order to standardize the assay method for routine analysis, an understanding of how physical and chemical factors affect the stability of the recombinant prion protein (rPrP) substrate and the RT-QuIC assay’s sensitivity, specificity, and reproducibility is required. In this study, using sporadic Creutzfeldt-Jakob Disease brain homogenate to seed the reactions and an *in vitro*-expressed recombinant prion protein, hamster rPrP, as the substrate, the following factors affecting the RT-QuIC assay were examined: salt and substrate concentrations, substrate storage, and pH. Results demonstrated that both the generation of the quality and quantities of rPrP substrate critical to the reaction, as well as the RT-QuIC reaction itself required strict adherence to specific physical and chemical conditions. Once optimized, the RT-QuIC assay was confirmed to be a very specific and sensitive assay method for sCJD detection. Findings in this study indicate that further optimization and standardization of RT-QuIC assay is required before it can be adopted as a routine diagnostic test.

## Introduction

Prion diseases, such as Creutzfeldt-Jakob Disease (CJD), are invariably fatal degenerative syndromes of the central nervous system. The central event of the disease process is the seeded conversion of host-encoded cellular prion protein (PrP^c^) into a misfolded disease-associated isoform (PrP^d^). Currently, definitive diagnoses rely upon the detection of PrP^d^ in a brain biopsy or *post mortem* brain tissue. The extremely low amounts of PrP^d^ present in other tissues make *ante mortem* tests based on its presence problematic.

One solution to this challenge has been the development of techniques that exploit the ability of PrP^d^ to seed the conformational conversion of a PrP^c^ substrate *in vitro*. Real-time quaking-induced conversion (RT-QuIC) is an example of a technique that utilizes minute amounts of PrP^d^ present in the test sample to seed the conformational conversion of normal soluble recombinant prion protein (rPrP^c^) from a highly α-helical structure into an amyloid fibril that is rich in β-sheet [1]. The technique has proven successful for sensitive prion detection in the 263K hamster scrapie model and in human CJD patient samples [2]–[5]. Utilizing brain homogenate (BH) or cerebral spinal fluid (CSF) samples, RT-QuIC has shown higher specificity than does 14-3-3 testing for sCJD [3], [6], [7] and specificity comparable to post-mortem immunohistochemistry of brain tissue [3], [6]. Hence, RT-QuIC has been shown to be a specific and sensitive method for PrP^d^ detection, with the potential to become the state-of-the-art *ante-mortem* clinical test for the diagnosis of sporadic Creutzfeldt-Jakob disease (sCJD) [2], [3].

Before clinical application of RT-QuIC can be widely adopted, the assay must be standardized for optimal analytical performance. Currently, users of the technique employ various buffer conditions and diverse fluorescence reading outputs [3], [6]. In addition, acidic pH as well as higher salt and detergent concentrations have been shown to either enhance RT-QuIC reactions or result in spontaneous aggregation of the recombinant protein substrate [3].

To establish optimal RT-QuIC reaction conditions in our laboratory a commercially available CJD brain homogenate (National Institute for Biological Standards and Control, NIBSC), previously confirmed by Western blot to contain high levels of proteinase K resistant PrP, was utilised as the test seed. This seed, in combination with an in-house generated recombinant full-length hamster PrP (rPrP) as the substrate, was used to explore the physical and chemical factors affecting RT-QuIC. The parameters of the optimal reaction conditions, such as salt, pH, and substrate concentrations, were determined, as well as the effects of elution method, dialysis conditions, and storage on substrate quality.

## Results

### Optimization of rPrP Purification Procedure

In order to increase the yield of rPrP, slight modifications to published methods were made [4], [5]. First, slower rPrP refolding was applied (approximately 18 hours on an HPLC system). Following refolding, the column was transferred to an FLPC system and isocratic elution (500 mM imidazole, 100 mM sodium phosphate and 10 mM Tris-HCl, pH 5.8), as opposed to gradient elution, was performed. The generated eluate appeared cloudy and filtration using a 0.22 µm syringe filter was difficult. Gel-staining demonstrated that the amount of rPrP collected in the syringe-filter filtrate was small compared to the large amount of rPrP in the eluate (data not shown). This suggested that the 0.22 µm filter was blocking the passage of rPrP aggregates. Using these conditions, the concentration of rPrP in the filtrate was too low to proceed with RT-QuIC trials. When 7.5 mL fractions of eluate were collected into tubes containing 2.5 mL of 10 mM sodium phosphate (pH 5.8), the eluate (final concentration: 375 mM imidazole, 77.5 mM sodium phosphate, and 7.5 mM Tris-HCl, pH 5.8) appeared clear and the mixture was easily filtered by a 0.22 µm filter before dialysis against 10 mM sodium phosphate (5.8). This step of immediately diluting the eluate was critical for the production of sufficient amounts of RT-QuIC compatible rPrP.

### Determination of Optimal RT-QuIC Conditions

In order to replicate previously published conditions [4], [5] rPrP was added to reaction mixtures that consisted of EDTA (1 mM), thioflavin T (ThT, 10 mM), and 1X phosphate buffered saline (10 mM sodium phosphate, 138 mM NaCl, and 2.7 mM KCl). In addition to the 138 mM NaCl contributed by PBS, many laboratories add additional NaCl to the reaction mixture, resulting in final NaCl concentrations that range between 138–500 mM [3]–[5]. When using similar substrate concentrations (10 µg/well) and reaction mixtures containing 50–200 mM NaCl in addition to the contribution from PBS, little change in ThT fluorescence was observed (Figure S1).

**Figure 1:**
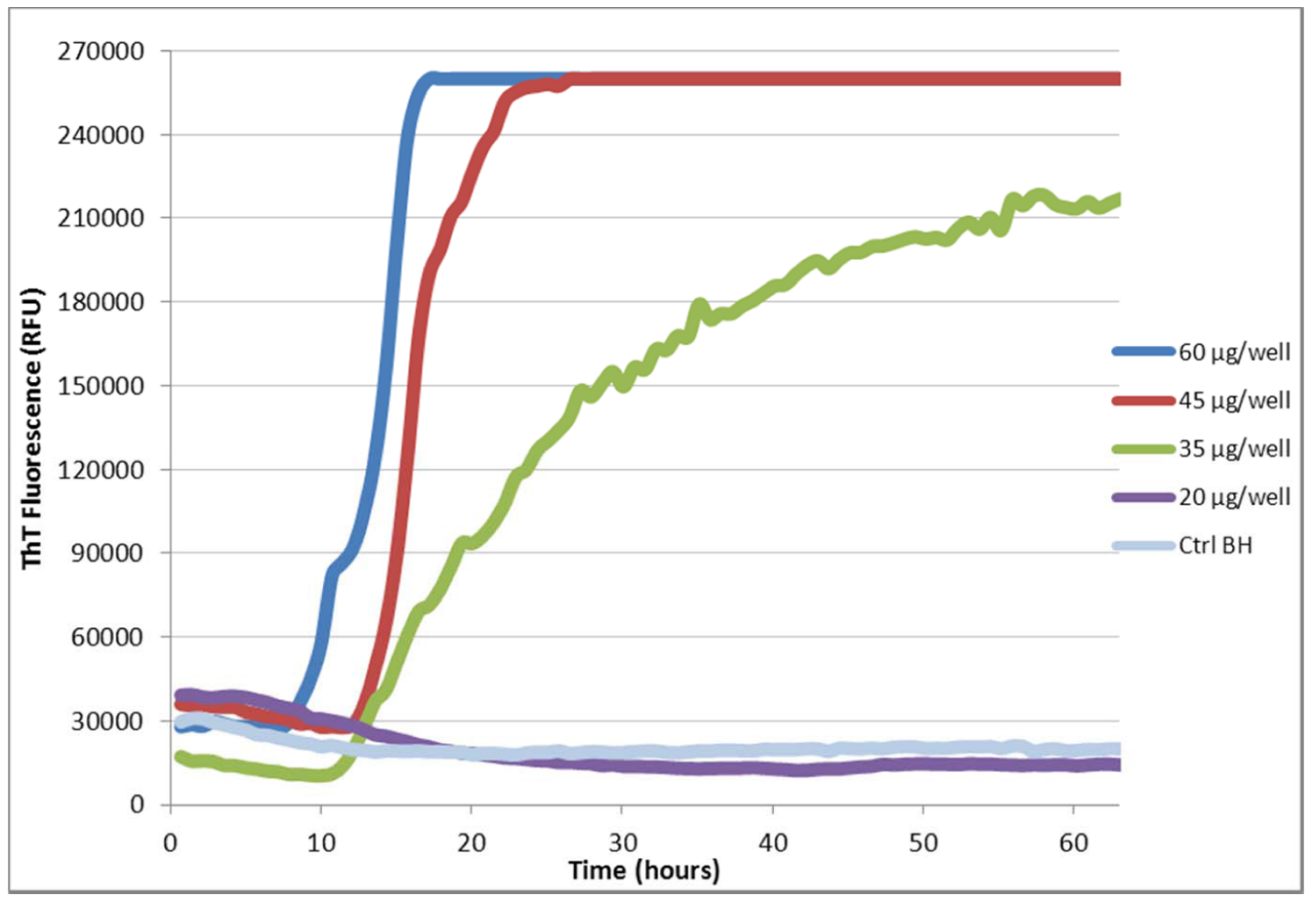
The effect of substrate concentration on RT-QuIC. RT-QuIC was performed using hamster rPrP substrate at concentrations of 20, 35, 45, and 60 µg/well. Reactions contained minimal salt (5.5 mM NaCl) and employed sCJD M/V brain homogenate (BH) diluted 10^−4^ to seed conversion.

**Figure 2:**
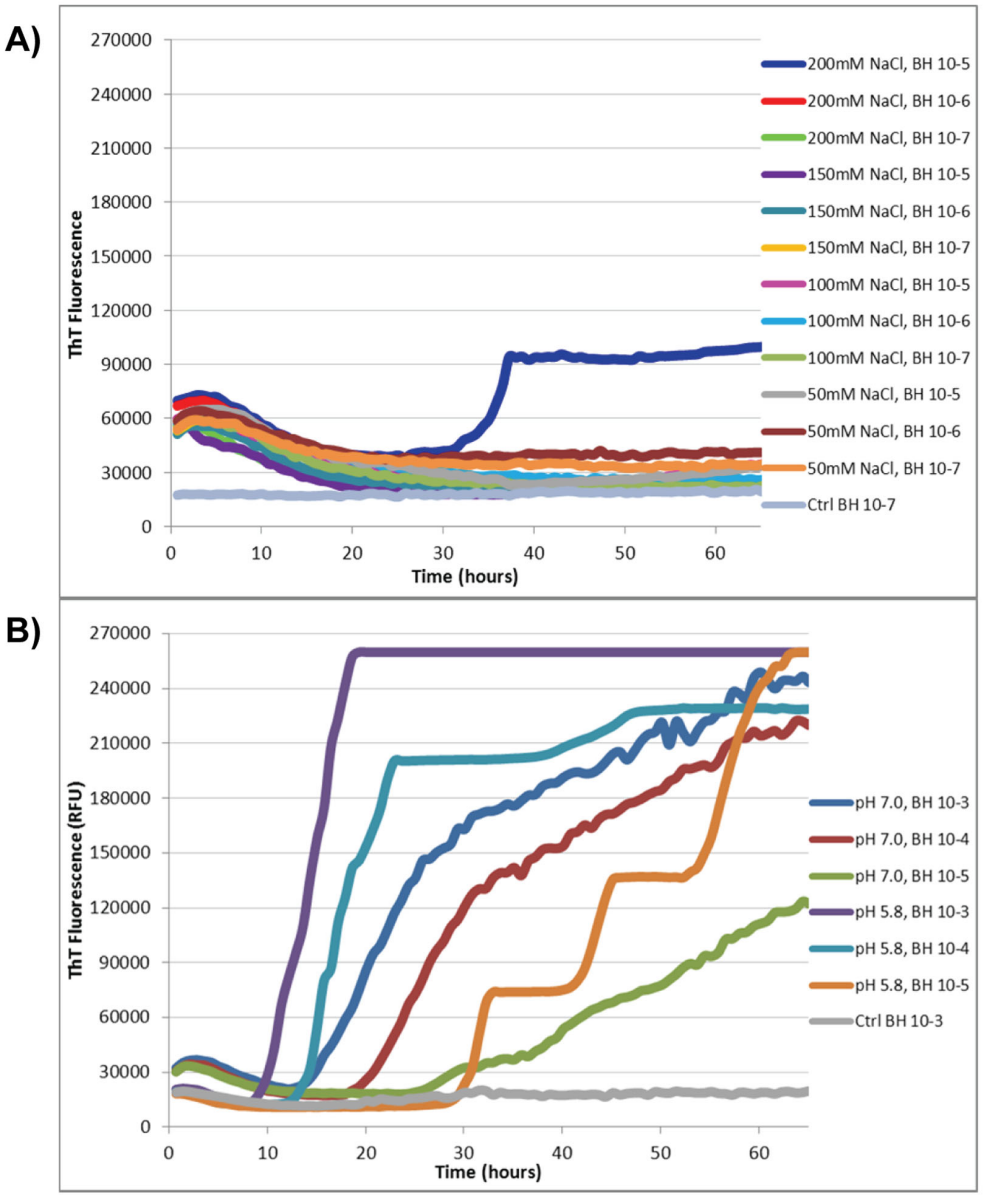
The effect of pH and salt on RT-QuIC when substrate concentration is 60 µg/well. (A) RT-QuIC was performed using reaction mixtures at pH 5.8 and 7.0 with minimal salt (5.5 mM NaCl). (B) RT-QuIC was performed using various concentrations of NaCl in the reaction mixture. PBS in the reaction mixture contributed 138 mM NaCl, and resulted in final salt concentrations of 188, 238, 288, and 338 mM where the additional NaCl concentrations were 50, 100, 150, and 200 mM, respectively. Reactions employed hamster rPrP at a concentration of 60 µg/well as the substrate and sCJD M/V brain homogenate (BH) at indicated dilutions to seed conversion.

It was noted in performing the above RT-QuIC experiments that the reaction mixtures became cloudy once a salt concentration of 238 mM was exceeded. This observation, and the difficulty encountered when trying to filter undiluted eluate, suggested that increased salt promoted rPrP precipitation, possibly rendering much of the substrate unavailable for conversion. To test this hypothesis, a variety of modified conditions using lower salt concentrations and increased amounts of substrate were investigated. A successful RT-QuIC reaction, indicated by a rapid increase in ThT fluorescence, was observed when the only NaCl present in the reaction mixture was from the PBS used to dilute the seed. The final NaCl concentration in these reaction mixtures was 5.5 mM. In addition, under these low salt conditions, the lag phase of the reaction, observed prior to the rapid conversion of rPrP to an amyloid form and increased thioflavin fluorescence, shortened as the amount of rPrP in the reaction mixture increased (Figure 1). The optimum conditions determined by this experiment were a final NaCl concentration of 5.5 mM and 60 µg/well of substrate. However, having altered two variables, both NaCl concentration and the amount of substrate, it was decided to test the effect of various salt concentrations on a reaction using a substrate concentration of 60 µg/well. The use of final salt concentrations as low as 50 mM failed to replicate the positive results seen with minimal salt (Figure 2A). It was noted that reactions that contained additional salt also had higher initial fluorescence prior to the start of the reaction. This may be indicative of aggregated rPrP prior to the start of seeded conversion.

### The Effect of Acidic pH on RT-QuIC Sensitivity

Previous studies have reported optimal reaction buffers of pH 5.8 and pH 7.0 [3], [4]. In both instances, these buffers contained considerably more salt than the concentration we found to be optimal for the RT-QuIC reaction. The effects of either pH 5.8 or pH 7.0 on an RT-QuIC assay employing 60 µg/well of hamster rPrP (23–231) as the substrate, sCJD BH as the seed, and a final concentration of 5.5 mM NaCl in the reaction buffer, were determined (Figure 2B). A determination of the final pH of the reaction buffer was not made, but as the bulk of the final reaction volume (96 µL of 100 µL) was made up of hamster rPrP, it was presumed that the pH of the reaction mixtures were approximately 5.8 and 7.0, respectively. At comparable concentrations of BH seed, the pH 5.8 reaction buffer showed increased sensitivity.

### rPrP Substrate Sensitivity to Temperature and Long-term Storage

Temperature was found to play an important role during rPrP purification. FPLC eluates containing higher rPrP concentrations became cloudy when placed on ice for several minutes, but reverted to optical clarity and were filtered easily upon return to room temperature. The substrate solution also became cloudy during dialysis at 4°C (pH 7.0), but remained clear at room temperature. These observations suggested that rPrP is prone to precipitation at lower temperatures. The aforementioned substrates worked as effectively in RT-QuIC assays as did those fractions that had not been refrigerated at any time during production (data not shown). Room temperature was nonetheless adopted as a condition of dialysis and substrate buffer preparation to produce soluble rPrP which worked effectively in RT-QuIC reactions at concentrations of 45–60 µg per well. In some laboratories, filtering rPrP with 100 kD molecular cut-off spin filters has been used immediately before RT-QuIC in order to remove potential aggregates which can cause spontaneous aggregation during the assay [5]. This step was found to be unnecessary with our rPrP, as false positive RT-QuIC reactions were not observed when using unfiltered substrate.

After purification, the substrate was divided into aliquots and stored in 10 mM phosphate buffer, pH 5.8 at −80°C. On the day of RT-QuIC experiments, frozen aliquots were incubated at 37°C with occasional gentle mixing to hasten thawing, and placed at room temperature before use in the assay. Long-term (≥6 months) storage at −80°C caused only a slight loss of sensitivity (Figure S2).

### Reproducible and Reliable RT-QuIC

**Figure 3:**
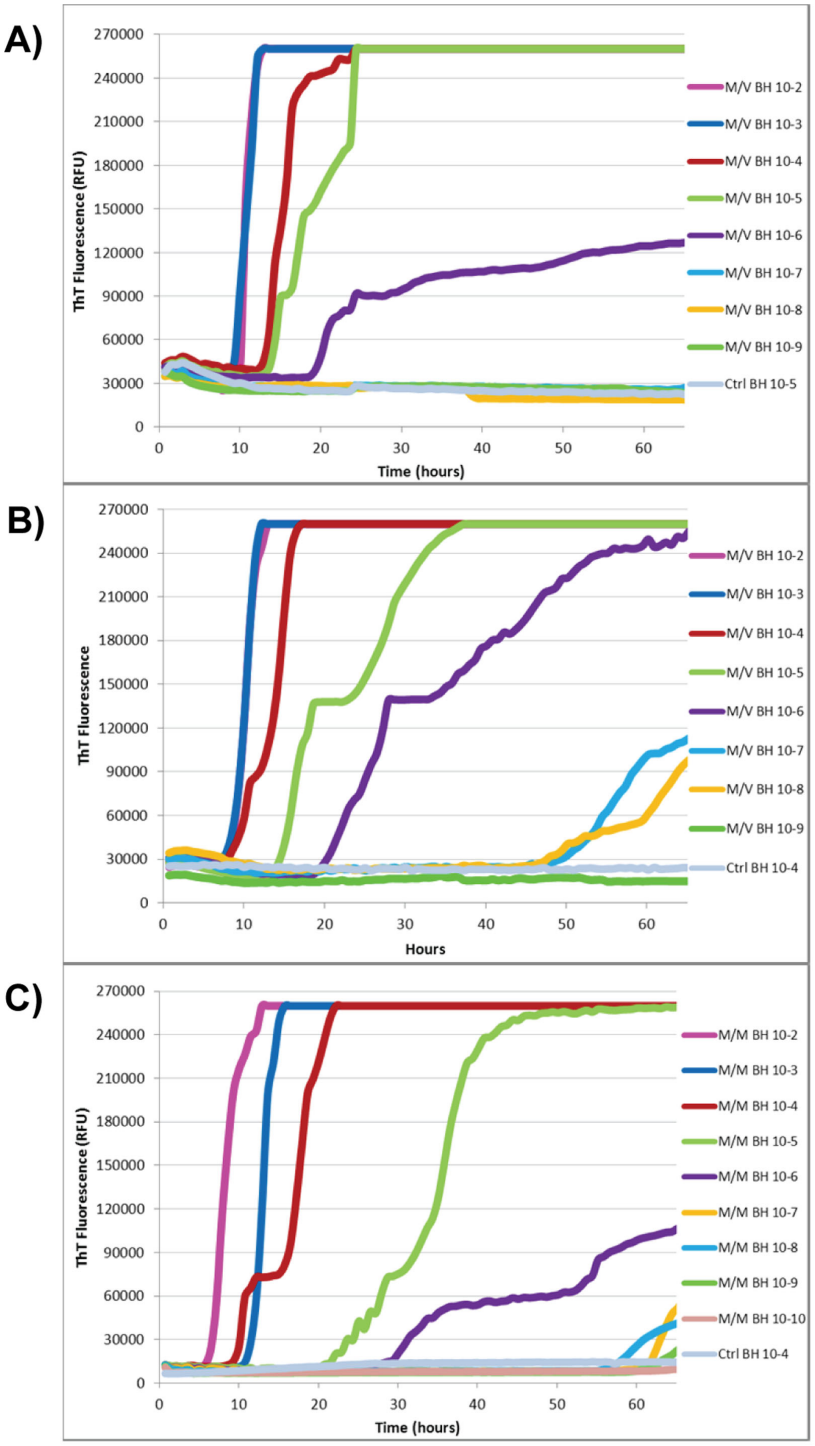
The effect of batch variability and seed genotype on RT-QuIC. RT-QuIC was performed using two different batches, Batch #1 (A) and Batch #2 (B), of hamster rPrP. Both assays employed hamster rPrP at a concentration of 60 µg/well as the substrate and sCJD M/V brain homogenate (BH) at the indicated dilutions as the seed. (C) RT-QuIC was performed using hamster rPrP Batch #1 at a concentration of 60 µg/well as the substrate and sCJD M/M brain homogenate (BH) at indicated dilutions to seed conversion. Reactions contained minimal salt (5.5 mM NaCl).

Upon establishing that the optimal conditions for our substrate and seed were minimal NaCl (5.5 mM), 60 µg/well of substrate, and pH 5.8, little test-to-test variability and rPrP batch-to-batch variability was observed (Figures 3A and 3B). A BH with an M/V genotype, indicating alleles encoding methionine and valine at codon 129 of the prion gene, was used in the aforementioned experiments because we were able to confirm by Western blot that it contained detectable levels of proteinase K resistant PrP. However, the M/M subtype is over-represented in the sCJD patient population. Therefore, it was of interest whether the conditions optimized for the M/V brain sample would work when tested using BH from MM cases. This analysis revealed that the two M/M samples from the same commercial supplier, having amounts of PrP^d^ below the level of detection by Western blot, produced strongly positive RT-QuIC results (Figure 3C and Figure S3). Though a commercially available homozygous V/V subtype sample could not be obtained an M/M variant CJD (vCJD) sample was available. This vCJD sample failed to promote seeded amyloid formation in our hands, a result that mimicked observations from other laboratories performing RT-QuIC (Figure S4) [5]. Therefore, in addition to little test-to-test and rPrP batch-to-batch variability, there was little seed-to-seed variability observed, indicating the robustness of the RT-QuIC assay using our substrate production and reaction conditions.

## Discussion

In this study, chemical and physical factors influencing RT-QuIC were assessed in order to promote the development of a standardized method. Assay optimization can vary with the species and strain of prion being assayed, the type of tissue sample, the sequence and storage conditions of the rPrP, and the relative priority given to assay speed versus sensitivity [3]–[6]; [8]–[10]. Here we have focused on the detection of PrP^d^ seeds in human sCJD brain homogenates using the full-length hamster rPrP substrate, which has been used extensively in other laboratories [4]–[6]. In our experience with this combination of prion source and rPrP, many elements needed to converge to produce a functional rPrP substrate, a key component for a successful RT-QuIC assay. First, rPrP should be diluted immediately after elution and remain in minimal salt during storage, reagent preparation, and throughout the assay. Second, rPrP should be kept at pH 5.8 if it is to be used for RT-QuIC. Third, working rPrP substrate solutions should be kept at room temperature to avoid precipitation. Moreover, thawing of frozen substrate should be performed at 37°C to minimize the length of time rPrP spends at lower temperatures, where precipitation most readily occurs. Fourth, 60 µg/well of substrate should be used for optimal sensitivity. Fifth, filtering rPrP with 100 kD molecular cut-off spin filters immediately before use in RT-QuIC may be unnecessary. We believe that our substrate was substantially free of aggregates due to slower refolding, producing a more thoroughly refolded product containing less residual guanidine-HCl. Lastly, rPrP can be stored for at least six months at −80°C and subsequently used in RT-QuIC trials with marginal effects on assay sensitivity. Whether or not the optimal conditions described for the RT-QuIC reaction, minimal salt, pH 5.8 and 60 µg/well of substrate will translate to other model systems or human CSF remains to be determined. Nonetheless, the discrepancy between the optimal salt and substrate concentrations reported here and those reported elsewhere are sure to promote continued debate and provide impetus towards continued efforts aimed at standardizing the RT-QuIC assay.

## Materials and Methods

### CJD and Control Brain Tissues

Brain homogenate samples from sCJD patients and non-sCJD control patients were purchased from the National Institute for Biological Standards and Control (NIBSC, reference codes NHBX0/0004 and NHBZ0/0005, respectively). They were prepared as 10 µl aliquots and stored at −80°C during RT-QuIC method development. The ethics statement found on the NIBSC website is appended below:

“NIBSC is licensed by the Human Tissue Authority (HTA) (Licence number 12321) to store human tissues for a limited range of specific purposes (known as scheduled purposes). Obtaining consent to remove, store, and use human tissues for specific purposes is one of the underlying principles of the Human Tissue Act and any samples of human tissue stored by NIBSC comply with this requirement of the act. When tissue samples are received at the NIBSC, they are retained securely and confidentiality and maintained in compliance with Caldicott principles, as are all samples received at this centre.”

Further information about the Human Tissue Act may be obtained from their website, http://www.hta.gov.uk/.

### Expression and Purification of RT-QuIC Substrates

*E. coli* possessing expression vectors containing full-length hamster rPrP (23–231) [4], [11], [12] was provided by Dr. Byron Caughey and his staff at the Rocky Mountain Laboratory in Hamilton, Montana. Recombinant PrP was expressed using the Overnight Express Autoinduction system (Novagen). Cell pellets from 1.5 liter cultures were frozen and thawed three times with liquid nitrogen and then lysed with two cycles of BugBuster Master Mix (Novagen) to isolate inclusion bodies. The inclusion bodies were then washed twice with 0.1X BugBuster, pelleted by centrifugation, and frozen at −80°C. Pellets were later resuspended and denatured in 8 M guanidine-HCl (pH 8.0) for 1.5 hours, centrifuged at 13,000×g for 15 minutes, and the supernatant bound to 50 ml of Ni-NTA Superflow resin (Qiagen) equilibrated in denaturing buffer (100 mM sodium phosphate, 10 mM Tris/HCl, 6 M guanidine-HCl, pH 8.0). The resin was incubated with the supernatant for 1.5 hours and loaded into a chromatography column (GE Healthcare XK-26). The denatured protein was refolded overnight (approximately 18 hours) using a linear gradient into refolding buffer (100 mM sodium phosphate, 10 mM Tris/HCl, pH 8.0) and flow rate of 0.8 ml/min on a Lab Alliance HPLC system (ThermoFisher). Bound protein was eluted using isocratic elution buffer (100 mM sodium phosphate, 500 mM imidazole, pH 5.8) at 2 ml/minute over 60 minutes on an AKTA FPLC system (GE HealthCare). Eluted protein was seeped into tubes containing 1/3 elution volume of dialysis buffer (10 mM sodium phosphate, pH 5.8), filtered with a 0.22 µm syringe filter, and dialysed at room temperature. The concentration of rPrP was determined by dividing its absorbance at 280 nm by its extinction coefficient (2.61 g^−1^ cm^−1^). The purity of rPrP was estimated to be 99% by SDS-PAGE and mass spectrometry (data not shown).

### RT-QuIC

RT-QuIC was performed as per Wilham et al. [4] and Peden et al. [5], with a few modifications. Frozen aliquots of rPrP were thawed at 37°C and kept at room temperature until they were added to the RT-QuIC master mix. The master mix was mixed gently and 96 µl was dispensed into the wells of a clear-bottomed black 96-well microplate (Nunc). After adding 4 µl of diluted brain homogenate to the wells, the final reactions contained 1/25X PBS (Sigma), 0.004% SDS, 0.04% N2, 1 mM EDTA, 10 mM ThT, and 0.6 mg rPrP per ml. Reactions were run in quadruplicate. The plates were sealed and inserted into a FLUOstar OPTIMA microplate reader (BMG Labtech), incubated at 45°C, and shaken intermittently (1 minute shaking, 1 minute at rest) at 700 RPM in a double orbital configuration. Fluorescence readings were taken at 480 nm every 45 minutes after excitation with 20 flashes per well at 450 nm. ThT emission fluorescence counts increased to a maximum of 260,000 per well during rPrP conversion. Graphical data was plotted as the mean of each quadruplicate test. A signal-to-noise ratio of 3∶1, as per International Conference of Harmonization (ICH) guideline [13] was considered a positive result.

## Supporting Information

Figure S1 **The effect of salt on RT-QuIC when substrate concentration is 10 µg/well.** RT-QuIC was performed using various concentrations of NaCl in the reaction mixture. PBS in the reaction mixture contributed 138 mM NaCl, and resulted in final salt concentrations of 188, 238, 288, and 338 mM where the additional NaCl concentrations were 50, 100, 150, and 200 mM, respectively. All reactions employed hamster rPrP at a concentration of 10 µg/well as the substrate and sCJD M/V brain homogenate (BH) at the indicated dilutions to seed conversion.
(TIF)

Figure S2 **The effect of freezing and storage of rPrP on RT-QuIC.** RT-QuIC was performed using Batch #1 hamster rPrP (60 µg/well) stored at −80°C for six months as the substrate. Reactions contained minimal salt (5.5 mM NaCl) and employed sCJD M/V brain homogenate (BH) at indicated dilutions to seed conversion. For comparison with results using fresh substrate, please see Figure 3A.
(TIF)

Figure S3 **The effect of seed genotype on RT-QuIC.** RT-QuIC was performed using hamster Batch #1 at a concentration of 60 µg/well as the substrate and a different sCJD M/M brain homogenate (BH; denoted as M/M-2 here) than that observed in Figure 3C, at the indicated dilutions. Reactions contained minimal salt (5.5 mM NaCl).
(TIF)

Figure S4 **The effect of seed disease pathology on RT-QuIC.** RT-QuIC was performed using hamster rPrP at a concentration of 60 µg/well as the substrate and vCJD M/M brain homogenate (BH) at indicated dilutions used to seed conversion. sCJD M/V brain homogenate was included as a positive control. Reactions contained minimal salt (5.5 mM NaCl).
(TIF)
