# Supplementary figures and images for: Exploring Physical and Chemical Factors Influencing the Properties of Recombinant Prion Protein and the Real-Time Quaking-Induced Conversion (RT-QuIC) Assay
Source: PLoS One. 2014 Jan 3;9(1):e84812. doi: 10.1371/journal.pone.0084812 (PMC3880330; doi:10.1371/journal.pone.0084812)

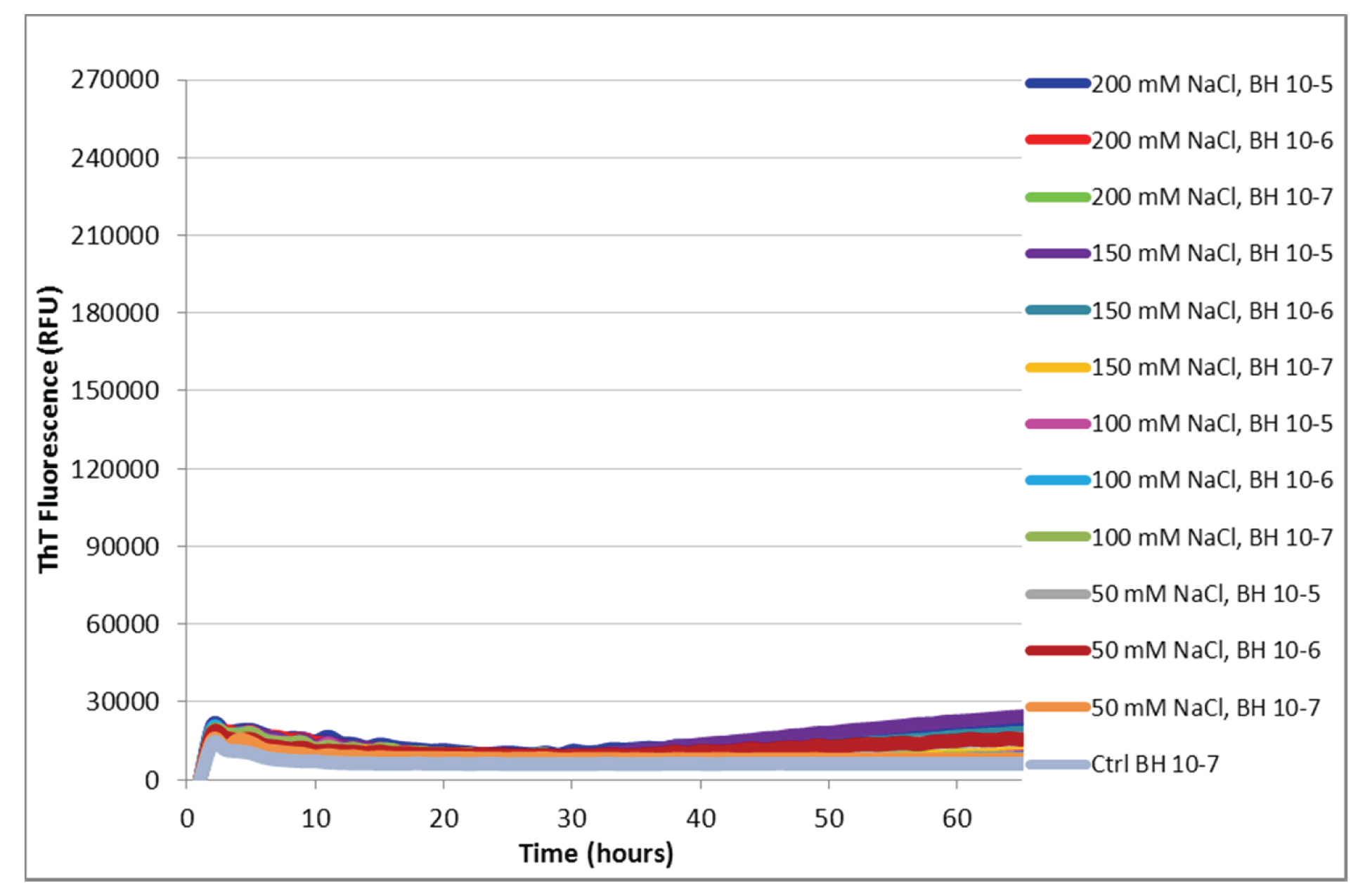

Supplement: Figure S1 — The effect of salt on RT-QuIC when substrate concentration is 10 µg/well. RT-QuIC was performed using various concentrations of NaCl in the reaction mixture. PBS in the reaction mixture contributed 138 mM NaCl, and resulted in final salt concentrations of 188, 238, 288, and 338 mM where the additional NaCl concentrations were 50, 100, 150, and 200 mM, respectively. All reactions employed hamster rPrP at a concentration of 10 µg/well as the substrate and sCJD M/V brain homogenate (BH) at the indicated dilutions to seed conversion. (TIF) [file pone.0084812.s001.tif]

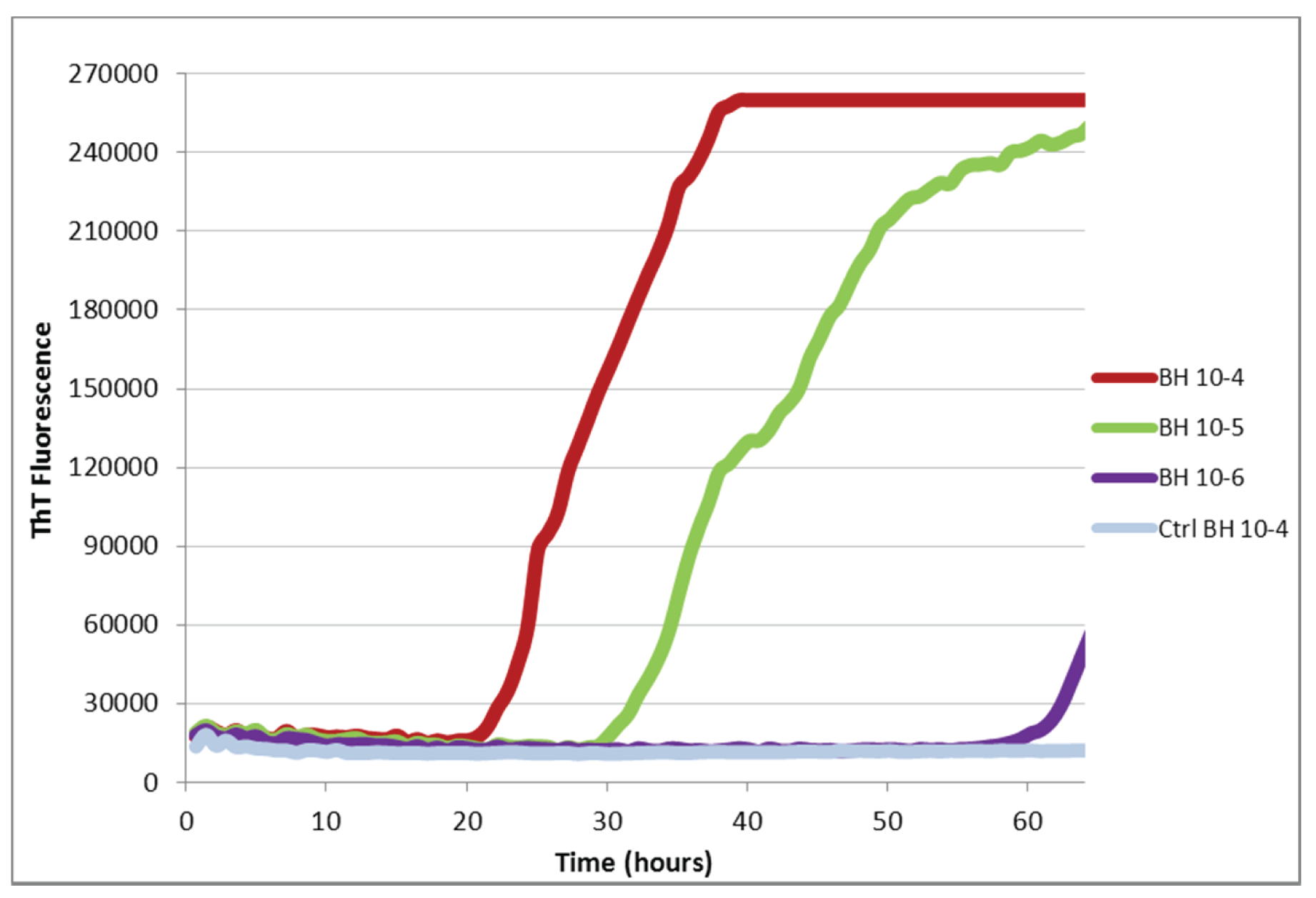

Supplement: Figure S2 — The effect of freezing and storage of rPrP on RT-QuIC. RT-QuIC was performed using Batch #1 hamster rPrP (60 µg/well) stored at −80°C for six months as the substrate. Reactions contained minimal salt (5.5 mM NaCl) and employed sCJD M/V brain homogenate (BH) at indicated dilutions to seed conversion. For comparison with results using fresh substrate, please see Figure 3A. (TIF) [file pone.0084812.s002.tif]

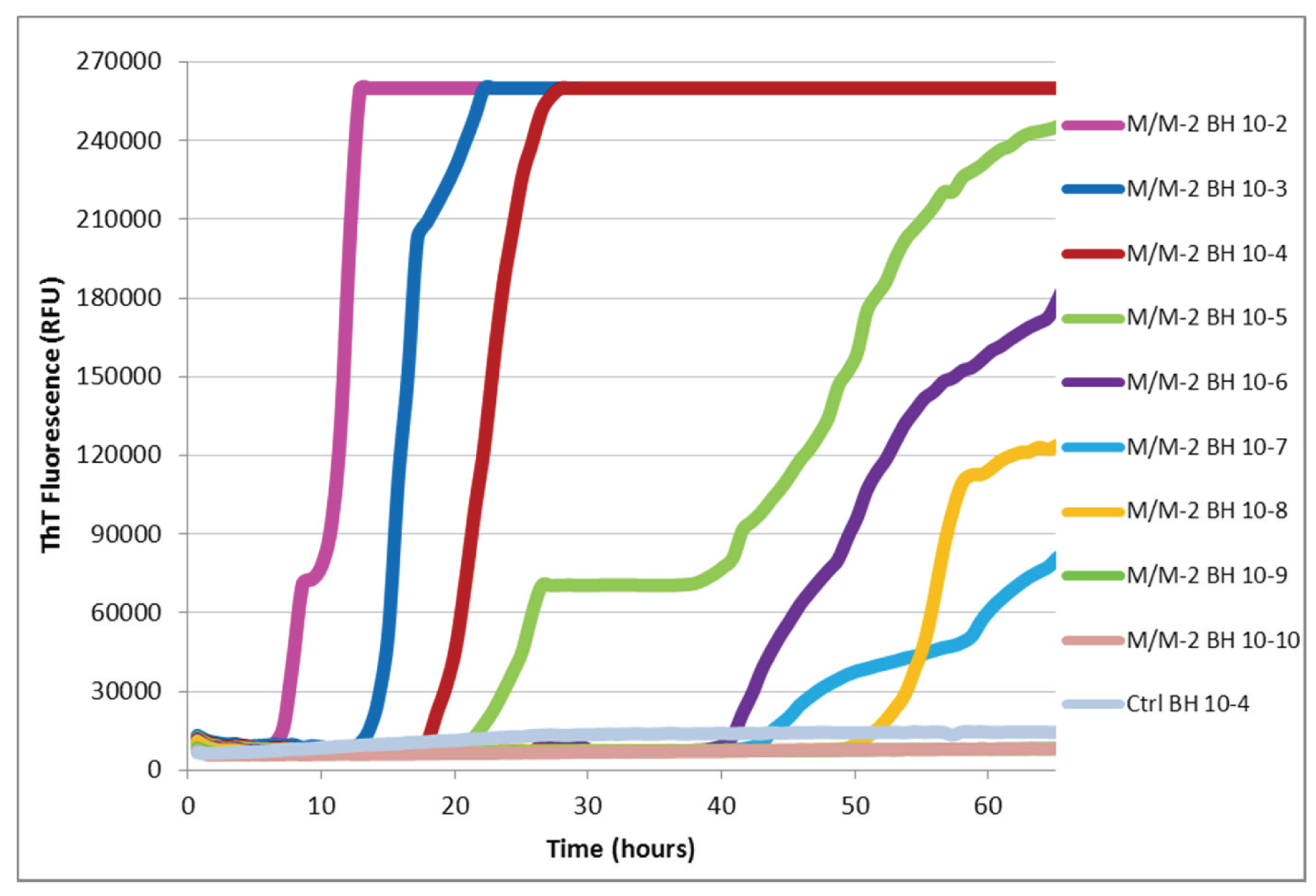

Supplement: Figure S3 — The effect of seed genotype on RT-QuIC. RT-QuIC was performed using hamster Batch #1 at a concentration of 60 µg/well as the substrate and a different sCJD M/M brain homogenate (BH; denoted as M/M-2 here) than that observed in Figure 3C, at the indicated dilutions. Reactions contained minimal salt (5.5 mM NaCl). (TIF) [file pone.0084812.s003.tif]

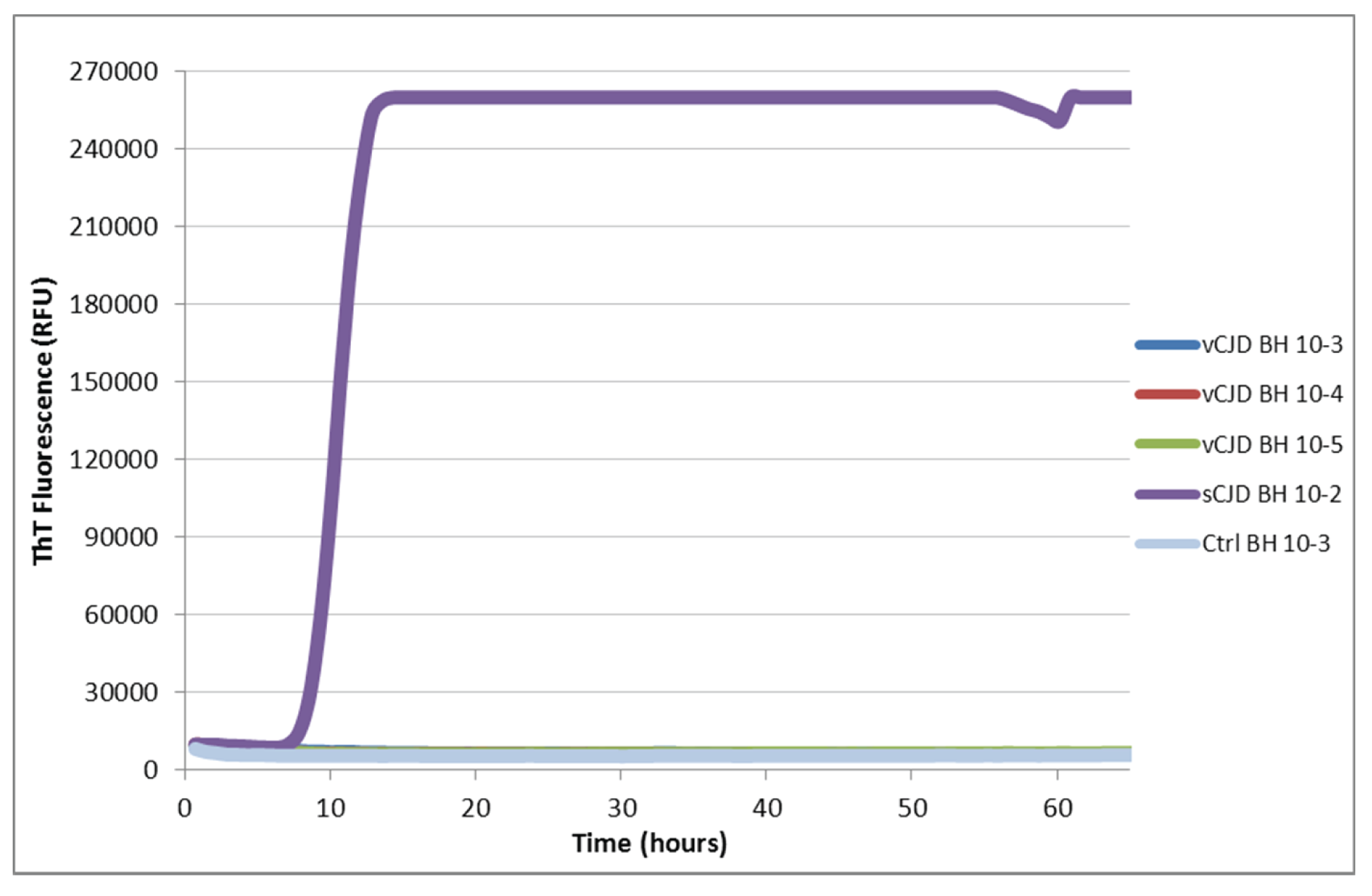

Supplement: Figure S4 — The effect of seed disease pathology on RT-QuIC. RT-QuIC was performed using hamster rPrP at a concentration of 60 µg/well as the substrate and vCJD M/M brain homogenate (BH) at indicated dilutions used to seed conversion. sCJD M/V brain homogenate was included as a positive control. Reactions contained minimal salt (5.5 mM NaCl). (TIF) [file pone.0084812.s004.tif]
